# Reviewed article: Oliveira TM, Araújo ACM, Costa GAT, Gomes LP, Alcantara TMPDC, Carvalho NS, Gusmão JD, Levi M, Burian APN, Ferraz ML, Silva TPR, Matozinhos FP. Social environment and services at the Special Immunobiological Reference Centers: epidemiological study, Minas Gerais, 2022-2024. Epidemiol Serv Saude. 2026:35;e20250092

**DOI:** 10.1590/S2237-96222026v35e20250092.a

**Published:** 2026-02-06

**Authors:** Cláudia Carolina Costa Braga

**Affiliations:** 1 Universidade Estadual Paulista Júlio de Mesquita Filho, Botucatu, SP, Brazil

## Peer review A

## Questionnaire

Does the manuscript contain new and significant information to justify publication? Yes

Does the Abstract (Summary) clearly and accurately describe the content of the article? Yes

Is the problem significant and concisely stated? No

Are the methods described comprehensively? No

Are the interpretations and conclusions justified by the results? Yes

Is adequate reference made to other work in the field? Yes

## Manuscript Structure

Length of article is: Adequate

Number of tables is: Adequate

Number of figures is: Adequate

Please state any conflict(s) of interest that you have in relation to the review of this paper. None

Interest: Good

Quality: Average

Originality: Good

Overall: Average

Recommendation: Major Revision

## Comments

- Descreva os métodos com mais detalhes, incluindo no campo “Contexto” informações quanto aos critérios utilizados pela Unidade regional de saúde para realizar os pedidos (como por exemplo, se devem atender aos critérios do manual dos CRIE). Essa informação é importante, pois o estudo discute o acesso aos serviços. O mesmo campo também descreve como é realizado o registro das solicitações, porém não informa os locais (Gerências/ Unidades Regionais) que podem solicitar os imunobiológicos ao CRIE-MG (alguns elementos estão descritos na discussão linha 52, página 5 da Discussão). Devido a limitação do número de palavras do manuscrito, se necessário, sugere-se descrever somente o modo do registro de solicitações que estava sendo realizado no momento do estudo. No campo “Mensuração” não descreve como foi realizada a tabulação de dados: se houve transcrição/organização dos dados secundários para outra planilha e qual o formato da planilha original disponibilizada (eletrônico, qual o programa, ou físico).

- Verifique a pertinência de incluir as seguintes palavras chave já existentes no DeCS e que melhor descrevem sua pesquisa: Programas de Imunização; Imunoglobulinas; Ambiente Social. Os descritores: Centro de Referência para Imunobiológicos Especiais; Imunobiológicos; Estudo ecológico não estão cadastrados no DeCS.

- Destaque a originalidade do trabalho, relacionando-o com a literatura existente.

É recomendável que seja considerado no texto que, para a prescrição de imunobiológicos especiais, é necessário que os indivíduos estejam dentro dos critérios de recebimento, conforme o Manual dos CRIE, relacionando com dados já publicados em literatura, de forma a enriquecer a discussão quanto aos achados da pesquisa.

- Descreva os procedimentos empregados no estudo para minimizar vieses, garantindo a validade e confiabilidade dos resultados. Nos métodos, o conteúdo do item “Controle de viés” está contemplando aspectos éticos; no campo dos “Participantes” não há descrição quanto aos critérios de exclusão dos registros quanto a duplicidade, solicitações com dados faltantes, etc, e nem quanto ao procedimento realizado para minimizar esses vieses.

- Explique as implicações práticas das descobertas, discutindo sua relevância para as politicas de saúde e futuras pesquisas. Justifique por que “Acredita-se que os resultados deste estudo contribuem diretamente para o fortalecimento dos CRIE e do PNI, incidindo na promoção e proteção da saúde coletiva”.

- O texto precisa ser revisado para garantir clareza, precisão e objetividade na linguagem. As idéias devem ser apresentadas de forma organizada e lógica. 1) Na Introdução: Página 2, linha 5: a ideia da frase permanece em aberto; Página 2, linhas 23 e 31: ajustar o tempo verbal quando se referir ao ano de 2024 (passado); Página 2, linha 33: há quebra de raciocínio entre as frases de um mesmo parágrafo; Página 3, linha 9: não traz elementos textuais que discorrem previamente sobre os fatores inerentes ao ambiente que influenciam os atendimentos não presenciais avaliados. 2) Nos Resultados e Discussão: deixar claro quando os dados se tratam das solicitações/atendimentos e quando se relacionam as prescrições realizadas. 3) Na página 7, linha 55 da “Discussão” não há referência quanto ao texto informado.

- A Tabela 1 trata-se de Quadro.

